# Application of on-demand aqueous chlorine dioxide solution for non-surgical root canal treatment

**DOI:** 10.1038/s41598-025-20131-5

**Published:** 2025-10-16

**Authors:** Tsuyoshi Shimaoka, Hazuki Maezono, Shunka Ono, Yoko Asahi, Yuzo Kawanishi, Kittipit Klanliang, Yusuke Takahashi, Mikako Hayashi

**Affiliations:** 1 Department of Restorative Dentistry and Endodontology, Graduate School of Dentistry, The University of Osaka, 1-8, Yamadaoka, Suita, Osaka 565-0871 Japan; 2 Department of Restorative Dentistry and Periodontology, Division of Endodontics, Chiang Mai University, Chiang Mai, Thailand

**Keywords:** Biofilm, Chlorine dioxide, MA-T, Root canal irrigation, Cytotoxicity, Osteoblast, Periodontal ligament fibroblast, Microbiology, Medical research

## Abstract

Oral biofilms contribute to apical periodontitis, a major cause of tooth loss. This study evaluated the potential of an on-demand aqueous chlorine dioxide solution, “matching transformation system” (MA-T), as a safer alternative to conventional root canal irrigants. The antibacterial activity of MA-T was assessed against mono-species biofilms (*Enterococcus faecalis*, *Parvimonas micra*, *Fusobacterium nucleatum*), polymicrobial biofilms derived from human supragingival plaque, and an in vitro infected root canal model using *E. faecalis*. Antibacterial efficacy was evaluated using viable cell counting and confocal laser scanning microscopy (CLSM) to quantify the reduction in bacterial load. Biofilm mass was assessed by crystal violet (CV) staining. Cytotoxicity was evaluated by treating rat osteoblasts and human periodontal ligament fibroblasts with MA-T or NaOCl, followed by assessment of ATP levels and morphological integrity. MA-T eradicated planktonic bacteria, inhibited biofilm formation, and significantly reduced viable bacteria in established biofilms without affecting the total amount of biofilm biomass time-dependently. The infected root canal model showed a substantial decrease in bacterial load. Compared with NaOCl, MA-T-treated cells retained higher ATP levels and normal morphology. MA-T could be a promising root canal irrigant offering effective disinfection and superior biocompatibility, making it a potential alternative to traditional root canal irrigants as NaOCl.

**Supplementary Information:**

The online version contains supplementary material available at 10.1038/s41598-025-20131-5.

## Introduction

The oral cavity contains more than 700 species of bacteria that form biofilm^1^, and they are the primary etiological agents of diseases, such as dental caries and apical periodontitis^2^. Dental caries result from bacteria-produced acids, which demineralize enamel and dentin, leading to pulpitis if left untreated, and carious lesions can progress to pulpitis and eventually lead to pulpal necrosis, which is a precursor of apical periodontitis of endodontic origin^3^. The infection then advances to apical periodontitis, an inflammation of the apical tissue that necessitates root canal treatment.

The goal of root canal treatment is to eliminate the existing infection and prevent microbial contamination of the root canal system^4^. Root canal treatment involves removing the infected pulp and dentin in the root canal^5^ by simultaneously performing thorough cleaning and disinfection^6^. This process includes mechanical enlargement of the root canal with dental instruments, such as endodontic files, and root canal irrigation using chemical agents to disinfect the inside of the root canal. Finally, the root canal is sealed with a filling material to prevent reinfection^7^. However, the disinfection process is challenging due to the presence of biofilms in the root canal system^8,9^. Microbial biofilms are embedded in self-produced extracellular polymeric substances (EPS)^10^, which enhance their resistance to antimicrobial agents and contribute to disease persistence and recurrence^11^.

Due to the complexity of the root canal system, it is technically impossible to remove all bacteria by mechanical removal alone^12^. Approximately 35% of the root canal surface remains untouched by instruments due to its intricate anatomy^13,14^. This complexity poses a significant challenge for effective disinfection and treatment, making it necessary to supplement mechanical enlargement with additional methods, such as the use of antimicrobial chemical solutions. It has been reported that irrigation with sodium hypochlorite (NaOCl) solutions can more effectively reduce the bacterial load within the root canal^6^.

NaOCl is used mostly as an irrigant during root canal treatment due to its strong antimicrobial and proteolytic activities; it is commonly used at concentrations ranging from 0.5% to 8.25%^15^. Though higher concentrations of NaOCl show enhanced bactericidal activity, the effectiveness may be affected by the pH of the solution. As NaOCl concentration increases, the pH rises, shifting the equilibrium toward OCl^–^, which is less effective than HOCl, the more active antimicrobial form. This shift could potentially reduce overall antimicrobial efficacy^16^. It has been reported that NaOCl can cause serious accidents, including facial nerve paralysis and necrosis, if extruded from the root apex^17^. These toxic effects are attributed to its alkalinity (pH 10.8 to 12.9) and hypertonicity, which can oxidize proteins and lipid membranes^18^. In some cases, these effects can lead to subcutaneous emphysema^19^. Despite these risks, NaOCl remains widely used due to its effectiveness and cost efficiency^20^.

However, many modern disinfectants face challenges in balancing efficacy and safety, and the development and application of disinfectants with high safety profiles have not progressed sufficiently. There is an increasing need for disinfectants in root canal treatment that are both highly effective and safe for patients. In this study, the focus was on a novel agent, an on-demand aqueous chlorine dioxide solution called “matching transformation system” (MA-T). MA-T generates aqueous radicals when bacteria are present^21,22^.

In addition, MA-T is not flammable, volatile, or corrosive; therefore, it is already used in medical care, including as a mouthwash, oral care gel, and as an environmental disinfectant^23^. When used as a mouthwash at a concentration of 100 ppm, MA-T has been reported to reduce oral bacteria, such as *Streptococcus mutans*^24^. However, previous studies have only evaluated the effects of MA-T against planktonic bacteria^24,25^, and there have been no reports on the impact of MA-T on oral biofilms.

The aim of the present study was to investigate the basic effects of MA-T on oral biofilms and host cells to examine its potential for application in endodontic treatment.

## Results

### Susceptibility to MA-T of planktonic bacteria related to endodontic infections

The minimum inhibitory concentrations (MICs) and minimum bactericidal concentrations (MBCs) of MA-T for *Enterococcus faecalis*, *Parvimonas micra*, and *Fusobacterium nucleatum* are shown in Table 1. The MICs ranged from 3.9 to 15.6 ppm, and the MBCs ranged from 3.9 to 31.3 ppm. Of the tested bacteria, *E. faecalis* exhibited the lowest MIC and MBC values, whereas *P. micra* had the highest.

**Table 1:** Minimum inhibitory concentrations (MICs) and minimum bactericidal concentrations (MBCs).

Bacteria strains	MIC	MBC
*Enterococcus faecalis* ATCC 29212	3.9 ppm	3.9 ppm
*Fusobacterium nucleatum* ATCC 23726	7.8 ppm	7.8 ppm
*Parvinomas micra* GIFU 7745	15.6 ppm	31.3 ppm

### Effects of MA-T on biofilm formation

**Fig. 1:**
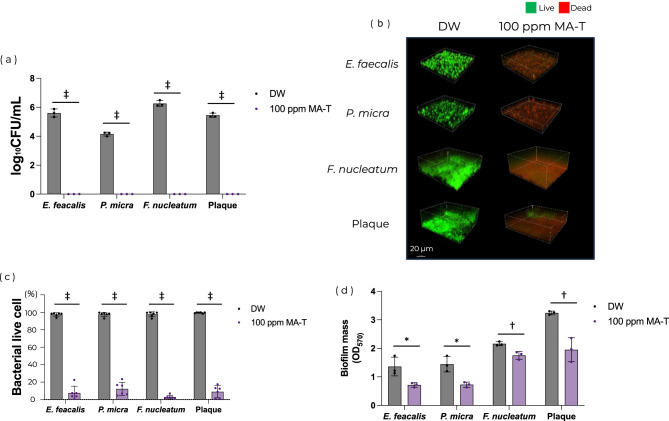
Effect of MA-T on biofilm formation. HA disks were incubated with bacterial culture or plaque suspension and then treated with MA-T. After application, biofilms were assessed using viable cell counts, and CLSM observation and crystal violet staining were performed. Data of (**a**) viable cell counting (n = 3), (**b**) representative LIVE/DEAD-stained CLSM images, (**c**) bacterial viability calculated from CLSM images (n = 6 per group) and (**d**) crystal violet staining (n = 3) are shown. Statistical analysis was performed using Student’s *t*-test (*P < 0.05, ^†^*P* < 0.01, ^‡^*P* < 0.001). All experiments were performed using biologically independent samples.

During biofilm formation, MA-T significantly inhibited the growth of each of the tested bacterial strains compared with the controls (*P* < 0.001; Fig. 1a). In the groups treated with MA-T, viable cell counts for all bacterial strains and plaque were completely eliminated, effectively preventing biofilm formation. Confocal laser scanning microscopy (CLSM) observations showed that over 90% of the bacteria in the control samples of each tested strain remained viable. In contrast, MA-T-treated samples showed a significant increase in dead bacteria, and less than 20% of the cells were viable (*P* < 0.001; Fig. 1b,c). In addition, crystal violet staining showed that MA-T significantly decreased biofilm mass and inhibited biofilm formation in all tested bacterial strains. Specifically, *E. faecalis*, *P. micra*, and plaque showed approximately 40% reductions, whereas *F. nucleatum* showed a 20% reduction. (*P* < 0.05; Fig. 1d).

### Effects of MA-T on established biofilms

**Fig. 2:**
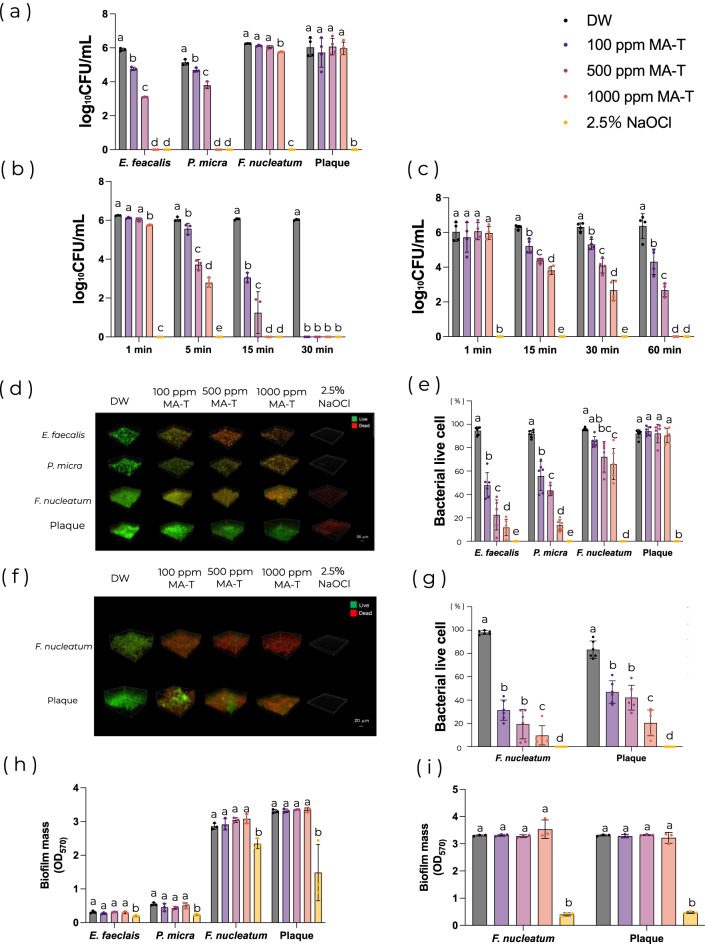
Effects of MA-T on established biofilms. (**a**) One minute of application: viable cell counting (n = 3 per group). (**b**) Effect on *F. nucleatum* biofilm with an extended application time: viable cell counting (n = 3 per group). (**c**) Effect on polymicrobial biofilm from human plaque: viable cell counting (n = 4 per group). (**d**) Representative LIVE/DEAD-stained CLSM images after 1 min of application and (**e**) bacterial viability calculated from CLSM images (n = 6 per group). (**f**) Representative LIVE/DEAD-stained CLSM images after 30 min of application and (**g**) bacterial viability calculated from CLSM images (n = 6 per group). (**h**) One minute and (**i**) 30 min of application: crystal violet staining. (n = 3 per group). Statistical analysis was performed using one-way analysis of variance (ANOVA) with Tukey’s honestly significant difference (HSD) post hoc test (*P* < 0.05). Different letters indicate a significant difference for each strain and application time. CLSM images were obtained from different samples within the same experimental group. All experiments were performed using biologically independent samples.

MA-T significantly reduced the number of viable bacteria in *E. faecalis* and *P. micra* biofilms compared with the controls after 1 min of application at all tested concentrations (*P* < 0.05; Fig. 2a). Viable bacteria counts for *E. faecalis* were 5.89 log_10_CFU/mL for the control, 4.77 log_10_CFU/mL for 100 ppm MA-T, and 3.09 log_10_CFU/mL for 500 ppm MA-T, and those for *P. micra* were 5.14 log_10_CFU/mL for the control, 4.69 log_10_CFU/mL for 100 ppm MA-T, and 3.8 log_10_CFU/mL for 500 ppm MA-T (Fig. 2a). For *F. nucleatum*, MA-T significantly reduced the number of viable bacteria after 5 min of exposure. Viable bacteria counts were 6.05 log_10_CFU/mL for the control, 5.57 log_10_CFU/mL for 100 ppm MA-T, 3.71 log_10_CFU/mL for 500 ppm MA-T, and 2.79 log_10_CFU/mL for 1000 ppm MA-T (*P* < 0.05; Fig. 2b). In the polymicrobial biofilms from supragingival plaque, MA-T significantly reduced the number of viable bacteria after 15 min of exposure (*P* < 0.05; Fig. 2c). CLSM observations showed that MA-T treatment for 1 min significantly increased the number of dead bacteria in *E. faecalis* and *P. micra* biofilms, with a reduction of more than 50% in bacterial viability (*P* < 0.05; Fig. 2d,e). In *F. nucleatum* and polymicrobial biofilms from plaque, MA-T significantly reduced the number of viable bacteria after 30 min of exposure, with more than a 50% decrease (*P* < 0.05; Fig. 2f,g). Overall, MA-T inhibited the biofilm bacteria in the established biofilms in an exposure time- and concentration-dependent manner (Fig. 2a–g). NaOCl reduced both the number of biofilm bacteria and biofilm mass volume (Fig. 2); in contrast, MA-T did not significantly affect biofilm mass volume at any of the tested concentrations (Fig. 2h,i).

### Effects of MA-T in the infected root canal model

**Fig. 3:**
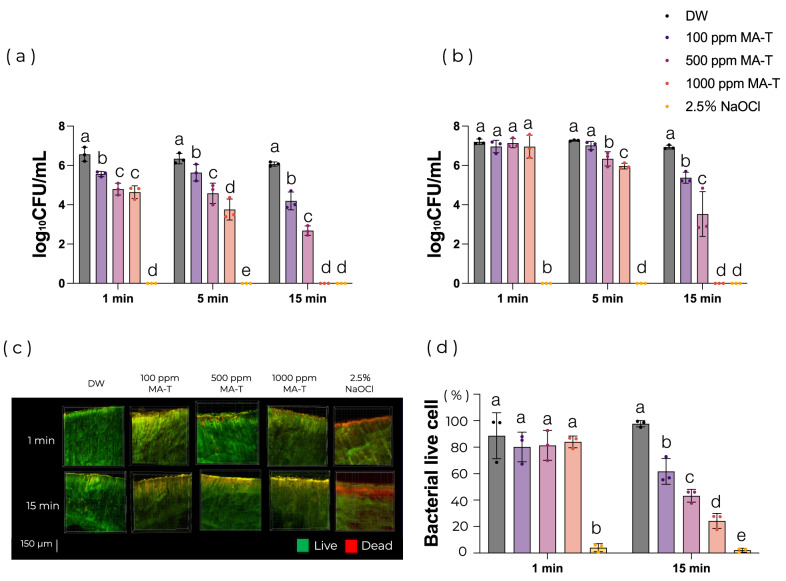
Effects of MA-T in the infected root canal model. (**a**) The paper point samples (n = 3 per group). (**b**) The circumferential filing samples (n = 3 per group). (**c**) Representative LIVE/DEAD-stained CLSM images. (**d**) Bacterial viability calculated from CLSM images (n = 3 per group). Statistical analysis was performed using one-way ANOVA with Tukey’s HSD post hoc test (*P* < 0.05). Different letters indicate a significant difference at each application time. CLSM images were obtained from different samples within the same experimental group. All experiments were performed using biologically independent samples.

Brown-Brenn staining confirmed that *E. faecalis* had penetrated up to 470 μm into the dentinal tubules in the infected root canal model using bovine teeth (Supplemental figure). Root canal irrigation with MA-T significantly reduced the number of bacteria in the paper point samples after 1 min of irrigation (*P* < 0.05; Fig. 3a). In the circumferential filing group, root canal irrigation for more than 5 min with 500 ppm MA-T (6.33 log_10_CFU/mL) showed a significant difference compared with the control group (7.26 log_10_CFU/mL) (*P* < 0.05; Fig. 3b). Root canal irrigation with 1000 ppm MA-T for 15 min showed a similar effect as 2.5% NaOCl in both groups (Fig. 3a,b). CLSM observation showed a significant reduction in the number of bacteria after 15 min of irrigation at all tested MA-T concentrations; in detail, 100 ppm MA-T resulted in a reduction of more than 30% in viable bacteria, whereas 1000 ppm MA-T led to a 70% reduction in viable bacteria (*P* < 0.05; Fig. 3c,d).

### Biological safety assessment of MA-T

**Fig. 4:**
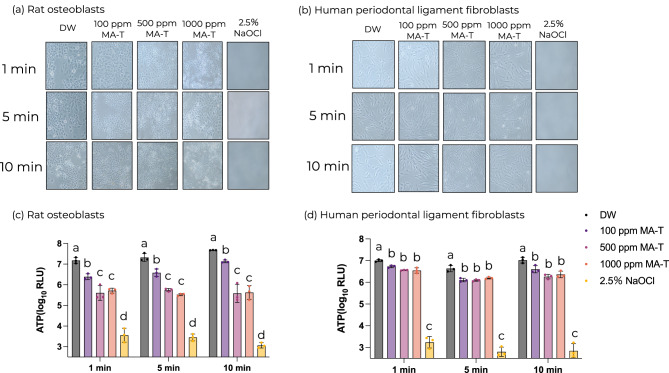
Biological safety assessment of MA-T. Representative images of (**a**) rat osteoblasts and (**b**) human periodontal ligament fibroblasts under an inverted microscope at 40 × magnification. Cellular ATP levels were measured after the application of MA-T. (**a**) Rat osteoblasts and (**b**) human periodontal ligament fibroblasts (**b**) (n = 3 per group). Statistical analysis was performed using one-way ANOVA with Tukey’s HSD post hoc test (*P* < 0.05). Different letters indicate a significant difference for each cell strain and application time. All experiments were performed using biologically independent samples.

No differences in cell morphology were detected in the MA-T-treated samples compared with the control samples, regardless of MA-T concentration (Fig. 4a,b). In contrast, cells treated with NaOCl showed cell lysis, and cell morphology could not be observed (Fig. 4a,b). In addition, the cell viability assay results showed that the MA-T-treated cells had significantly lower ATP levels than the control cells, but significantly higher ATP levels than the NaOCl-treated cells. In particular, MA-T caused a 20% decrease in ATP levels in osteoblasts and an 8% decrease in hPDLFs. In contrast, NaOCl treatment resulted in a greater than 60% reduction in ATP levels in both osteoblasts and hPDLFs (*P* < 0.05; Fig. 4c,d).

## Discussion

In this study, the effects of a novel disinfectant, MA-T, were evaluated for its antibacterial activity against bacteria and its antibiofilm effects in oral biofilm samples, in an in vitro infected root canal model, as well as its cytotoxicity on osteoblasts and hPDLFs to assess its potential for application in endodontic treatment. The results showed that MA-T effectively killed planktonic bacteria, prevented biofilm formation, and reduced the number of bacteria in infected root canals. Importantly, MA-T was found to be less toxic to host cells than NaOCl solution, a commonly used disinfectant.

In comparison with commonly used root canal irrigants such as NaOCl, chlorhexidine (CHX), and ethylenediaminetetraacetic acid (EDTA), MA-T seems to show both antimicrobial efficacy and biocompatibility, offering important advantages. NaOCl remains the gold standard due to its strong tissue-dissolving and bactericidal properties, but its high cytotoxicity and risk of damaging periapical tissues remain significant concerns^17^. CHX is less cytotoxic than NaOCl and exhibits substantivity, but it lacks tissue-dissolving ability and has limited efficacy against biofilm-embedded bacteria^26^. EDTA is primarily used to remove inorganic smear layers and does not possess sufficient antimicrobial activity^20^. Hypochlorous acid (HOCl) has been explored as a low-toxicity alternative, but its instability and short shelf-life limit its clinical utility^27^. However, direct effects of CHX, EDTA, and HOCl compared with MA-T were not examined in the present study, and further studies are needed for detailed comparisons.

The process of biofilm formation can be divided into three stages: planktonic bacteria, bacterial attachment to solid surfaces, and matured biofilm embedded in EPS^28^. To comprehensively assess the anti-biofilm efficacy of MA-T, its effects were evaluated in all three stages of biofilm formation. In the first stage, the MICs and MBCs of MA-T in planktonic bacteria were similar for all tested bacterial strains, indicating that MA-T is highly effective as an antibacterial agent. In the second stage, MA-T was found to have an inhibitory effect on biofilm formation. In the third stage, it is important to evaluate the state of the bacteria within the biofilm to assess the efficacy of antimicrobial materials.

Viable cell counting is a standard method for quantifying the number of bacteria. However, this method cannot identify bacteria in a ‘viable but non-culturable’ (VBNC) state^29^, where cells remain metabolically active but incapable of growth due to external stressors or environmental conditions. Since VBNC bacteria can grow again if the environment changes to one with optimal conditions^30^, we cannot underestimate their potential as a source of infection. CLSM observation enables assessment of the three-dimensional structure and state of the bacteria inside biofilms, including VBNC bacteria. However, CLSM tends to lead to overestimations of the number of dead bacteria^31^. In this study, viable cell counting and CLSM observations were combined, because these two methods can compensate for each other’s weaknesses and allow comprehensive assessment of biofilm bacteria. Notably, CLSM successfully enabled the detection of bacteria that were not detected by viable cell counting, which is consistent with findings from previous studies^30,31^. Combining viable cell counting and CLSM observations appears to be necessary for evaluating the state of bacteria in biofilms and the efficacy of antimicrobial materials.

Regarding the effects of MA-T on established biofilms, the time before an antimicrobial effect on biofilm bacteria could be detected differed for each bacterial strain; this may be due to differences in biofilm structures. Crystal violet staining showed that the total biofilm volumes of *F. nucleatum* and plaques were more than five times larger than those of the other bacterial species tested in this study. *Fusobacterium* species have a larger bacterial cell size (3 to 10 μm in diameter) than the other bacterial species tested in this study, which may have resulted in a larger biofilm. Human plaques are composed of various bacterial species^32^ and generate a higher quantity of extracellular polysaccharides than mono-species biofilms^33^. Consequently, MA-T may require a longer duration to penetrate and exert its antibacterial effect on polymicrobial biofilms from plaque than the mono-species biofilms evaluated in this study. In addition, since only the total amount of biofilm was quantified using crystal violet staining in the present study, further studies on each component of biofilms, such as EPS, are needed.

The bactericidal mechanism of MA-T has been reported to involve disruption of the bacterial respiratory chain^25^. Chlorine dioxide, the active component of MA-T, is a strong oxidizing agent that targets various bacterial constituents, including amino acids such as tyrosine and tryptophan, nucleic acids, and membrane lipids^34^. Through this broad oxidative mechanism, MA-T exerts bactericidal effects independently of bacterial respiration, and it is therefore considered effective even against strictly anaerobic bacteria that rely on fermentation. In addition, chlorine dioxide can penetrate biofilms without disrupting the EPS^35,36^, enabling direct inactivation of bacteria residing within the biofilm.

Chlorine dioxide has been reported to increase the permeability of both outer and cytoplasmic membranes, resulting in the leakage of essential intracellular components such as nucleic acids, which are strongly associated with loss of cellular activity or cell death^37^. In contrast, transmission electron microscopy (TEM) images have been reported to show no obvious morphological damage or cell lysis^37^, suggesting that the biofilm mass itself remains largely unchanged. These findings are consistent with those of the present study, in which MA-T was shown to act on biofilm-embedded bacteria without disrupting the overall biofilm structure.

An in vitro infected root canal model using bovine teeth infected with *E. faecalis* was used in this study. Although it would be ideal to prepare an infected root canal model using human teeth, bovine teeth were used to standardize the samples, including root length and morphology. Bovine teeth are reported to have a similar morphology, physical properties, and chemical composition to human teeth^38,39^. In the present study, *E. faecalis* was found to have penetrated dentinal tubules to a depth of 470 μm (Supplemental figure). In a previous study, *E. faecalis* was found to invade human dentinal tubules to a depth of 420 μm^40^, similar to the present result.

*E. faecalis* was used as a source of infection in the infected root canal model because it is frequently detected in refractory apical periodontitis and is a representative bacterium of chronic apical periodontitis^41,42^. However, in clinical settings, apical periodontitis is a polymicrobial infection in the oral cavity^43^, and this infected root canal model could not reproduce the complexity of an actual infected root canal. In the future, it will be necessary to establish an ex vivo multi-strain-infected root canal model to clarify the effects of MA-T.

In the infected root canal model, paper point samples and circumferential filing samples were used to determine the viable bacterial count. The method of collecting bacteria in the root canal using paper points has been applied for the evaluation of the infection status in both clinical practice and experimental settings^42,44^. However, this paper point method can only collect planktonic bacteria in the root canal, and the number of bacteria collected varies depending on the insertion position^42,44^. Therefore, in addition to collecting bacteria using paper points, the bacteria that had invaded into dentinal tubules of the same tooth were also evaluated by collecting chips during circumferential filing of the root canal. In the paper point samples, the amount of bacteria was significantly lower in the samples treated with MA-T for 1 min than in the control samples; however, in the circumferential filing samples, a significant reduction in the number of bacteria was observed only in the samples treated with MA-T for 15 min. Therefore, the collection of bacteria using only the paper point method may lead to an overestimation of the effect of root canal irrigants.

As with the viable cell count results, CLSM observations in the infected root canal model also showed an increasing amount of dead bacteria as irrigation time increased, indicating that MA-T has a time-dependent bactericidal effect on the bacteria in the dentinal tubules it also demonstrated the discrepancy between detection methods. From the results of the present study, it appears that the analysis of circumferential filing samples and CLSM observation are the most appropriate methods for evaluating the bacteria in the root canal.

In a rat model of apical periodontitis, apical lesions healed when the number of bacteria in the root canal was reduced by more than 75% during root canal treatment^45^. In the present study, root canal irrigation with 100 ppm MA-T for 15 min reduced the number of bacteria in the root canal by more than 75%, which is considered to promote healing. Although a MA-T application time of 15 min is rather long for syringe irrigation, it may be clinically feasible as a working solution during mechanical cleaning and root canal enlargement, since the average working time for root canal enlargement has been reported to be 30 min^6,46^. In addition, a longer MA-T application time resulted in a stronger anti-biofilm effect in the present study, suggesting that MA-T could possibly be used not only as a root canal irrigant, but also as a root canal medicament if used with a longer application time.

Regarding the safety of MA-T, it has been reported that oral administration of 100 to 3000 ppm of MA-T in mice for 1 month had no effect on survival rates^47^, and no adverse effects were observed in a clinical study using 100 ppm of MA-T as an oral care gel^23^. Chlorine dioxide, the main ingredient in MA-T, is considered safe, and it is used as a water disinfectant. In the present study, the biosafety of MA-T was tested on osteoblasts and periodontal ligament cells. MA-T application did not affect the morphology of the cells; in contrast, NaOCl caused cell lysis. The ATP levels were lower in the MA-T-treated cells than in the control cells, but the levels were significantly higher than those in the NaOCl-treated cells. Chlorine dioxide has been reported to damage cellular DNA and affect the cell cycle without altering cell morphology^48^. In the present study, the observed decrease in ATP levels without changes in cell morphology suggests that MA-T may have damaged cellular DNA. Previous reports have also indicated that DNA damage in cells leads to a decrease in ATP levels^49^.

The present study demonstrated that MA-T is effective in killing planktonic bacteria, inhibiting biofilm formation, and reducing bacteria within established biofilms, while maintaining lower cytotoxicity than NaOCl. These findings suggest that MA-T could be a promising alternative to conventional irrigants in endodontic treatment, especially when used as a working solution during root canal treatment. Further studies aiming for actual clinical application are needed.

## Material and methods

### Bacterial strains and culture conditions

Two Gram-positive coccus strains, *Enterococcus faecalis* ATCC 29212 and *Parvimonas micra* GIFU 7745, and a Gram-negative bacillus strain, *Fusobacterium nucleatum* ATCC 23726, were used to prepare mono-species biofilms. These bacteria have been frequently isolated from infected root canals^50^. *E. faecalis* ATCC 29212 and *F. nucleatum* ATCC 23726 were purchased from the American Type Culture Collection. The *P. micra* GIFU 7745 used in the present study was isolated from infected root canals^51^.

*E. faecalis* was cultured in brain–heart infusion (BHI) broth (Becton Dickinson, Sparks, MD, USA). *P. micra* and *F. nucleatum* were cultured in Gifu anaerobic medium broth (Nissui, Tokyo, Japan) containing 1% hemin (Sigma-Aldrich, St. Louis, MO, USA) and 0.02% vitamin K3 (FUJIFILM Wako Pure Chemical Corporation, Osaka, Japan). Each bacterial strain was incubated from the stock culture for 12 h at 37 °C under anaerobic conditions using AnaeroPack-Anaero (Mitsubishi Gas Chemical Company, Inc., Tokyo, Japan).

Ex vivo polymicrobial biofilms from plaque were prepared according to the method of Maezono et al.^52^ Briefly, supragingival and subgingival plaque samples from molar and premolar regions were obtained with sterilized wooden sticks from four volunteers (two males and two females aged 26 to 42 years (mean age, 31.5 ± 7.1 years)) who had no systemic or oral abnormalities and had not received any antibiotics or other medications within the past 6 months, and the suspension was used immediately after collection.

The experiments using human dental plaque were conducted with the approval of the Ethics Committee Review Board of Osaka University Graduate School of Dentistry (approval no. H30-E25, R6E-3). The procedures used in the present study complied with the tenets of the Declaration of Helsinki, and all volunteers provided written, informed consent to participate in the study.

### Antimicrobial susceptibility testing

The MICs of MA-T against planktonic bacteria were determined to range from 0 to 1000 ppm by the standard method of the Japanese Society of Chemotherapy^53^ using 96-well U-bottom microplates (Becton Dickinson).

Specifically, 100 µl of serially diluted test agent in medium and 100 µl of bacterial suspension was added to each well was added to each well of a 96-well round-bottom tissue culture plate, and bacterial inoculation was prepared at a final concentration of approximately 10^6^ colony-forming units (CFU)/well. The plate was then incubated anaerobically for 24 h for *E. faecalis* and 48 h for *P. micra* and *F. nucleatum*. The MIC was determined as the lowest concentration of the test agent in which no visible bacterial growth was observed. After determining the MIC, the culture liquid from wells showing no turbidity was collected and seeded onto BHI agar plates. The MBC was defined as the lowest concentration at which no colony formation was observed on the agar plate after anaerobic incubation, as for MIC determination*.* The experiment was performed independently three times.

### Effects of MA-T on biofilm formation

Hydroxyapatite (HA) discs (8.9 mm in diameter and 1.6 mm in thickness; BONECERAM; Olympus Terumo Biomaterials, Tokyo, Japan) were used as the substrate for biofilm formation. The HA discs were treated with bovine dermal type I collagen solution (10 μg/mL collagen and 0.012 N HCl in water; Cohesion, Palo Alto, CA, USA) for 8 h for surface treatment, as in our previous study^52^. Optical density (OD) was adjusted as follows for preparing the bacterial suspensions: for *E. faecalis*, OD_405_ = 0.05^54^; for *P. micra* and *F. nucleatum,* OD_600_ = 0.1^55^; and for human supragingival plaque*,* OD_405_ = 0.1^56^. The collagen-treated HA disks were incubated in a bacterial culture solution or plaque suspension at 37 °C for 3 days under anaerobic conditions. Subsequently, MA-T was then added to the culture medium at a concentration of 200 ppm to achieve a final concentration of 100 ppm, then incubated in the presence of MA-T for another 4 days to prepare biofilms. Control specimens were exposed to sterile distilled water for 4 days instead of the MA-T solutions. After application of the MA-T, the biofilms were rinsed twice with sterile distilled water (Otsuka Pharmaceutical, Tokyo, Japan). The biofilms were evaluated by viable cell counting, CLSM observations, and crystal violet staining, as described below. All data were obtained from biologically replicated experiments.

### Effects of MA-T on established biofilms

For preparing biofilms, collagen-treated HA disks were incubated with bacterial culture solutions or plaque suspension under anaerobic conditions at 37 °C for 7 days. The biofilm samples were rinsed twice in 2 mL of sterile distilled water, then treated with 2 mL of 100, 500, or 1000 ppm MA-T, or 2.5% NaOCl solution (Neocleaner, Neo Dental Chemical Products, Tokyo, Japan). The different concentrations of MA-T solutions were freshly prepared by diluting a 1000-ppm stock solution in sterile distilled water. Control samples were treated with sterile distilled water. After application of the MA-T or NaOCl solutions, the biofilms were rinsed twice, then evaluated by viable cell counting, CLSM observations, and crystal violet staining. All experiments were performed using biologically independent samples.

### Viable cell counting

Established biofilms of *E. faecalis* and *P. micra* were treated with MA-T or NaOCl for 1 min, those of *F. nucleatum* were treated for 1, 5, 15, or 30 min, and biofilms derived from human plaque were treated for 1, 15, 30, or 60 min. Based on differences in susceptibility observed among the bacterial species, treatment durations were adjusted accordingly. For species that showed lower responsiveness to MA-T in preliminary testing, longer exposure times were used to ensure adequate antimicrobial assessment.

After treatment, biofilms on the HA discs were detached by vortexing each disc in 10 mL of medium for 30 s. The resulting bacterial suspensions were then serially diluted. *E. faecalis* and plaque-derived samples were plated on BHI agar, and *F. nucleatum* and *P. micra* samples were plated on Gifu anaerobic medium agar supplemented with 5 µg/mL hemin and 1 µg/mL menadione. All plates were incubated anaerobically for 48 h, followed by CFU counting. The results are expressed as log_10_ CFU/mL (n = 3).

### CLSM analysis of biofilms

Established biofilms of *E. faecalis*, *P. micra,* or *F. nucleatum* and plaque biofilms were treated with MA-T or NaOCl for 1 min. In addition, the *F. nucleatum* and polymicrobial biofilms from plaque were also treated with MA-T or NaOCl for 30 min.

MA-T-treated samples were rinsed with distilled water, then stained using the LIVE/DEAD Bacterial Viability Kit (BacLight; Invitrogen, Carlsbad, CA, USA) for 15 min in the dark at room temperature. The samples were then observed by CLSM (LSM700; Carl Zeiss, Oberkochen, Germany).

CLSM was used to obtain z-stack images from the top to the bottom of each biofilm. The images were reconstructed and analyzed in three dimensions using Imaris software (version 9.2.1; Bitplane, Zurich, Switzerland). The fluorescence volumes corresponding to live (green) and dead (red) bacteria were calculated to assess bacterial viability. The ratio of live cells was determined by dividing the volume of green fluorescence by the total volume of both green and red fluorescence. For each condition, the center field of the disc was captured from each disc, resulting in a total of six independent fields analyzed per group (n = 6).

### Crystal violet staining

In the same way as for CLSM observation, biofilms on HA discs were rinsed twice with sterile distilled water to remove planktonic bacteria, then stained with 0.1% crystal violet solution (Sigma Aldrich) for 20 min to detect the biofilm mass. After staining, the biofilms on the HA discs were rinsed twice, then extracted with 95% ethanol. The OD was measured at 570 nm using a plate reader (ARVO MX-fla; Perkin Elmer, Waltham, MA, USA; n = 3).

### Effects of MA-T in the infected root canal model

#### In vitro infected root canal model and MA-T application

**Fig. 5:**
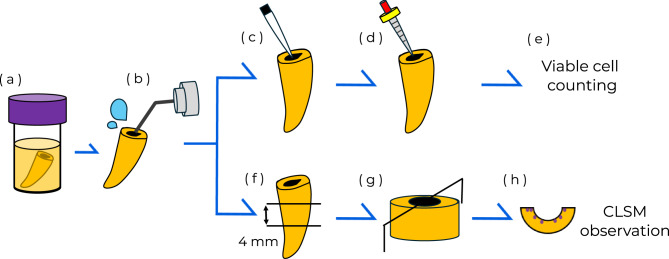
Experimental protocol for the root canal infection model. (**a**) Sterilized bovine teeth were immersed in *E. faecalis.* (**b**) Root canal irrigation using MA-T or NaOCl. (**c**) Collection of root canal bacteria using paper points (paper point samples). (**d**) The root canal was filled with sterilized distilled water, followed by circumferential filing (circumferential filing samples). (**e**) Viable cell counts were determined from paper point samples and the collected dentin tips. (**f**) Preparation of a dentin block, a longitudinal section. (**g**) Vertical sectioning of the dentin block. (**h**) Observation of the bacteria in dentinal tubules using CLSM.

The in vitro infected root canal model was prepared using bovine anterior teeth (Osaka Nanko Zouki, Osaka, Japan) and *E. faecalis* according to the method of Li et al. (Fig. 5)^57^. After removing the tooth crown, the length of the root was adjusted to 12 mm, and the root canal was enlarged using a #120 K-file (MANI, Tochigi, Japan). After root canal preparation, the canals were cleaned with 3 mL of 2.5% NaOCl solution using a 30-gauge side-vented irrigation needle (Shanghai International Holding, Shanghai, China). The samples were placed in a 3% EDTA solution (Smear Clean; Nippon Shika Yakuhin, Yamaguchi, Japan) for 1 min in an ultrasonic bath (UT-306; Sharp, Tokyo, Japan).

To avoid external microbial contamination, the external surface of the root was coated with two layers of nail varnish (Ida laboratories, Tokyo, Japan). The specimens were subsequently sterilized in an autoclave (LBS-245; TOMY, Tokyo, Japan) before bacterial infection.

The bovine teeth were then immersed in a suspension of *E. faecalis* adjusted to 10^7^ CFU/mL, and incubated anaerobically at 37 °C for 4 weeks to prepare an infected root canal. The BHI medium was refreshed every 3 days. The infection status was confirmed following the method of Kuremoto et al.^58^ using Taylor’s Brown-Brenn staining. Specifically, dentin blocks were fixed in a 4% paraformaldehyde-phosphate-buffered solution (Fujifilm Wako Pure Chemical) and 10% glutaraldehyde (Fujifilm Wako Pure Chemical) and then decalcified for 3 days in CalciTox (Fujifilm Wako Pure Chemical). After decalcification, the blocks were dehydrated through an ascending alcohol series, embedded in paraffin, and continuous sections of 8-μm thickness were prepared using a rotary microtome (RM 2155; Leica, Wetzlar, Germany). The sections were stained with crystal violet solution for 2 min, followed by staining with 2% Gram iodine solution (Fujifilm Wako Pure Chemical) for 1 min, then washed with water, dried, and decolorized by immersion in ether-acetone solution (Fujifilm Wako Pure Chemical). Subsequently, the sections were reacted with basic fuchsin solution (bioMérieux, Marcy, France) for 3 min, washed with water, treated with acetone-picric acid solution (Fujifilm Wako Pure Chemical), cleared in xylene (Fujifilm Wako Pure Chemical), and mounted. The infection status within the dentinal tubules was observed under an optical microscope (BZ-X700; KEYENCE, Osaka, Japan).

For the treatments, root canals were irrigated with 100, 500, or 1000 ppm of MA-T or 2.5% NaOCl solution for 1, 5, or 15 min. Root canal irrigation was performed at a flow rate of 3 mL/min. The total volume of irrigant used was 3 mL for the samples treated for 1 min, 15 mL for the samples treated for 5 min, and 45 mL for the samples treated for 15 min. Sterile distilled water was used for the control samples. After root canal irrigation, the samples were rinsed with 2 mL of sterile distilled water, then evaluated by viable cell counting and CLSM observations (Fig. 5). All experiments were performed using biologically independent samples.

#### Viable cell counting in the infected root canal model

After adding 300 μL of distilled water, bacteria in the root canal were collected using #80 sterile paper points (VDW, Munich, Germany) and placed in 10 mL of BHI medium. After sample collection using the paper points, 200 μL of sterile distilled water was poured into the root canal, and circumferential filing was performed using a #60 H-File (MANI). Filing debris and the remaining sterile distilled water in the root canal were transferred into 800 μL of sterile distilled water using a micropipette (Thermo Fisher Scientific, Waltham, MA, USA) and examined as circumferential filing samples. Both the paper point and circumferential filing samples were serially diluted and incubated anaerobically on BHI agar plates for 48 h. The resulting bacterial colonies were counted, and the results are expressed as log_10_ CFU (n = 3).

### CLSM observations in the infected root canal model

After 1 or 15 min of root canal irrigation, dentin blocks were prepared by excising the central 4-mm segment from a 12-mm root using a razor blade (Feather, Gifu, Japan) and a mallet. The dentin blocks were then split vertically into halves to prepare samples for observation.

After the dentin blocks were rinsed with sterile distilled water, they were stained using the LIVE/DEAD BacLight Bacterial Viability Kit for 15 min at room temperature. Subsequently, three randomly selected cross-sections from each specimen were observed by inverted CLSM (LAX R with NSPARC; Nikon, Tokyo, Japan). Data analysis was performed using image analysis software (Imaris 9.2.1.), and the proportion of viable bacteria was also calculated (n = 3).

## Biological safety assessment of MA-T

### Cell culture and MA-T application

Rat osteoblasts (UMR-106; Funakoshi, Osaka, Japan) and human periodontal ligament fibroblasts (ScienCell Research Laboratories, Carlsbad, CA, USA) were cultured in minimum essential medium α (α-MEM; Nacalai Tesque, Kyoto, Japan) containing 1.0 g/L glucose, 10% fetal bovine serum (Thermo Fisher Scientific), and 1% penicillin–streptomycin solution (FUJIFILM Wako Pure Chemical) at 37 °C under an atmosphere with 5% CO_2_, and the medium was changed every 3 days.

When the cultured cells were semiconfluent in the cell culture dishes (IWAKI, Tokyo, Japan), they were washed with Dulbecco’s phosphate-buffered saline (D-PBS; Thermo Fisher Scientific), then treated with a trypsin and EDTA solution (TrypLE Select; Thermo Fisher Scientific) for 3 min. After centrifugation at 240 × *g* for 5 min, the cells were resuspended in 1 mL of α-MEM, then counted using an automated cell counter (Countess 3 FL; Thermo Fisher Scientific).

For morphology observations, cells were seeded at 1.0 × 10^4^ cells/well in 6-well tissue culture plates (IWAKI) and cultured for 5 days under an atmosphere with 5% CO_2_. For the cell viability assay, cells were seeded in 96-well plates (Corning, Corning, NY, USA) at 1.0 × 10^4^ cells/well and incubated for 24 h. After removal of the medium, the cells were rinsed with D-PBS, then treated with 100, 500, or 1000 ppm of MA-T, or 2.5% NaOCl solution for 1, 5, or 10 min. The cells were then rinsed again with D-PBS and used in the following experiments. The control samples were treated with sterile distilled water. All experiments were performed using biologically independent samples.

### Cell morphology observations

After 3 mL of α-MEM was added into the 6-well tissue culture plates containing the treated cells, cell morphology was observed under an inverted microscope (TS100; Nikon) at 40 × magnification (n = 3).

### Cell viability assay

One hundred microliters of sterile distilled water and 100 μL of ATP measurement reagent (Cell Titer-Glo; Promega, Madison, WI, USA) were added to the cells and allowed to react for 15 min in the dark at room temperature. The amount of ATP released from the viable cells was measured using a multiplate reader (GloMax Explorer Multimode Microplate Reader; Promega; n = 3).

### Statistical analysis

IBM SPSS Statistics software (IBM, Chicago, IL, USA) was used for the statistical analyses. The Shapiro–Wilk test was used to confirm that the obtained data were normally distributed. Student’s *t*-test was used to compare two groups, and one-way analysis of variance and Tukey’s honestly significant difference post hoc test were used to compare multiple groups. A *P* value < 0.05 was considered significant.

## Supplementary Information

Supplementary Information.

## Data Availability

The datasets used in the current study are available through the corresponding author upon reasonable request.
