# Supplementary material for: Application of on-demand aqueous chlorine dioxide solution for non-surgical root canal treatment
Source: Sci Rep. 2025 Oct 16;15:36215. doi: 10.1038/s41598-025-20131-5 (PMC12533024; doi:10.1038/s41598-025-20131-5)
Supplement: Supplementary file 1 — Supplementary Information. [file 41598_2025_20131_MOESM1_ESM.pdf]

(a)

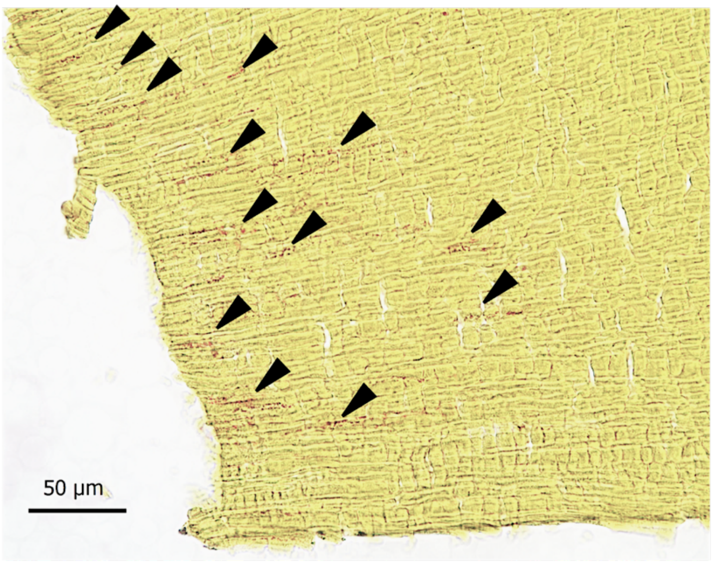

(b)

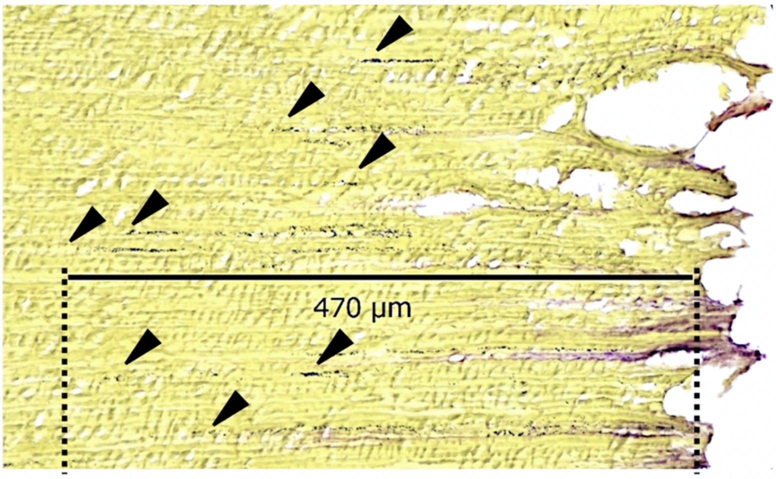

Supplemental figure

Histological evaluation for the construction of an *in vitro* infected root canal model using Brown–Brenn staining. (a) Lower maginification at ×20. (b) Higher maginification at ×40. (Arrowhead) *E. faecalis* within dentinal tubules.
